# ﻿Diversity of beetles (Arthropoda, Insecta, Coleoptera) associated with coniferous forests in Honduras

**DOI:** 10.3897/zookeys.1226.136987

**Published:** 2025-02-06

**Authors:** Mauricio Hernández, Mauricio Michel, Joel García, Geisy Dueñas, Marcela Moncada, Kevin Amaya, Yensi Yánez, Alejandra Pinto, Gabriela Matamoros, Alejandro Zamora, Gustavo Fontecha

**Affiliations:** 1 Instituto de Investigaciones en Microbiología, Facultad de Ciencias, Universidad Nacional Autónoma de Honduras, Boulevard Suyapa, Tegucigalpa, M.D.C., Honduras; 2 Escuela de Biología, Facultad de Ciencias, Universidad Nacional Autónoma de Honduras, Boulevard Suyapa, Tegucigalpa, M.D.C., Honduras; 3 Instituto Nacional de Conservación y Desarrollo Forestal, Áreas Protegidas y Vida Silvestre, Comayagüela, M.D.C., Honduras

**Keywords:** Ambrosia beetles, bark beetles, Coleoptera, Honduras, *Ipsapache*, pine forests

## Abstract

Bark beetles are among the primary drivers of tree mortality in coniferous forests worldwide. Individuals belonging to the order Coleoptera were identified across different forest areas in Honduras. Descriptive statistics were used to calculate the number of families, subfamilies, genera, and species collected per department. Moreover, the barcoding approach was used by amplifying and sequencing the mitochondrial COI gene. The intraspecific genetic diversity of *Ipsapache* was also analyzed. 1,131 individuals were examined and 27 genera were identified. Most of the specimens were identified as belonging to the genus *Ips*, accounting for 53.2% of the total. *Xyleborus* accounted for 16.5% and *Temnoscheila* accounted for 10%. Fewer than four individuals were found for fifteen genera. 68% of the specimens were identified to the species level, and all the specimens were identified to the genus level. *Ips*, *Temnoscheila*, *Xyleborus*, *Hypothenemus*, and *Pityophthorus* exhibited the most extensive geographic distribution among the sampled sites. At the genus level, Olancho, El Paraíso, and Copán displayed the highest diversity. This study also marks the first report of the genera *Xylomeira* and *Stephanopachys* in Honduran pine forests. Within *I.apache*, evidence of intraspecific genetic diversity was observed, although no population structure was detected. While this research provides an updated inventory of beetle species associated with Honduran coniferous forests, further taxonomic surveys and ecological studies are essential to better understand the spread and impact of bark beetles in pine ecosystems.

## ﻿Introduction

In the last decades, the faunistic composition of forest ecosystems has experienced significant changes because of bark beetle attacks (Morris et al. 2018). It has been estimated that bark beetle pests affect 30 million hectares of forest annually (FAO 2010). Consequently, they represent a substantial burden in terms of both economics and ecology due to their negative impact on conifer forests worldwide. Bark beetles (Coleoptera: Curculionidae, Scolytinae) comprise a large and diverse group of insects with more than 6,000 species (Wood 2007; Hulcr et al. 2015) distributed in all continents except Antarctica (Wood 2007). Although only a few genera are recognized as significant disturbance agents in coniferous forests, invasive species pose a substantial threat to native biodiversity (Smith and Hulcr 2015; Kirkendall 2018). Their presence can lead to tree mortality, ultimately disrupting the productivity and dynamics of forest ecosystems (Raffa et al. 2008). Further, several biological and mobility drivers can favor the risk of introduction and establishment of invasive species, increasing their geographic expansion (Grégoire et al. 2023). In addition, warmer temperatures and long-lasting droughts have been linked to tree stress, but also bark beetle survival and population growth (Breshears et al. 2005; Raffa et al. 2008), contributing to changes in tree physiological processes (e.g. leaf water potential, water content, osmotic potential) and greater coniferous forest infestation (Ryan et al. 2015).

Several bark beetle species have been the subject of research in Central America, particularly those in the genus *Dendroctonus* due to their aggressive behavior in invading and destroying pine forests (Billings et al. 2004; Armendáriz-Toledano et al. 2014; Gomez et al. 2020). Honduras, located in the geographic center of the Central American isthmus, is characterized by mountainous regions, from which coniferous forests cover approximately 1,951,977 ha, representing 30.91% of forest ecosystems (Hernandez et al. 2012; ICF 2022). These forests consist primarily of *Pinusoocarpa* and *P.caribaea* (Rivera-Rojas et al. 2010), playing a key role in many environmental and economic services, including habitat provision, genetic resources, water filtration, climate regulation, carbon sequestration, and erosion control, among others. However, some studies have also reported the detrimental effects of beetle outbreaks in Honduran coniferous forests (Rivera-Rojas et al. 2010; Valdez-Vasquez et al. 2020), leading to incalculable ecological and economic impacts. For instance, the Honduras Forest Institute of Conservation (ICF) reported that coniferous-dominated forests were extremely affected by *Dendroctonusfrontalis* between 2014–2016, a period characterized by prolonged droughts and low precipitation. Furthermore, the ongoing outbreaks of *Ips* beetle infestations have initiated ecological succession in Honduran pine forests, potentially causing cascading effects throughout forest ecosystems (ICF 2017). Given that bark beetle assemblages have profound ecological effects, it is critical to assess their taxonomic and spatial distribution to adequately mitigate beetle propagation, overall forest health, and resiliency.

In North and Central America, Wood´s monograph (Wood 1982) has been widely used for insect identification based on morphological features. However, species identification based on dichotomous keys often does not allow a precise identification at a lower taxonomic level due to a lack of information for new genera or species. Additionally, DNA barcoding analyses have been largely implemented to validate morphological identification in insect taxonomic inventories (Tahir et al. 2018; Wu et al. 2021; Zhang et al. 2022), including the order Coleoptera (Ho et al. 2022; Kodada et al. 2022; Thorn et al. 2022; Ren and Zhang 2024). As a result, integrating morphological and molecular methods enables accurate identification in taxonomic surveys and allows the assessment of population structure. Despite its broad relevance in taxonomic studies, the Honduran insect fauna has rarely been investigated by applying molecular techniques, highlighting some previous reports in thrips (Rugman-Jones et al. 2006), flies (Mejia et al. 2018; Orozco 2018), and mosquitoes (Escobar et al. 2020). Moreover, bark and ambrosia beetles, as well as their biological predators have never been evaluated using DNA barcoding analysis in Honduras. Consequently, our analyses comprise the first attempt to apply DNA barcoding to identify insect species associated with pine forests. In this study, we assess the biodiversity of beetles found in coniferous forests throughout different regions in Honduras. Additionally, due to secondary bark beetles of the genus *Ips* cause significant damage in coniferous forests (Negrón et al. 2009) and the actual status distribution is poorly known in our country, we also determined the spatial distribution and genetic diversity of *Ipsapache* beetle assemblages by analyzing intra-specific variation in the mitochondrial gene COI (cytochrome C oxidase subunit I).

## ﻿Materials and methods

### ﻿Study area and collection of the specimens

**Figure 1.**
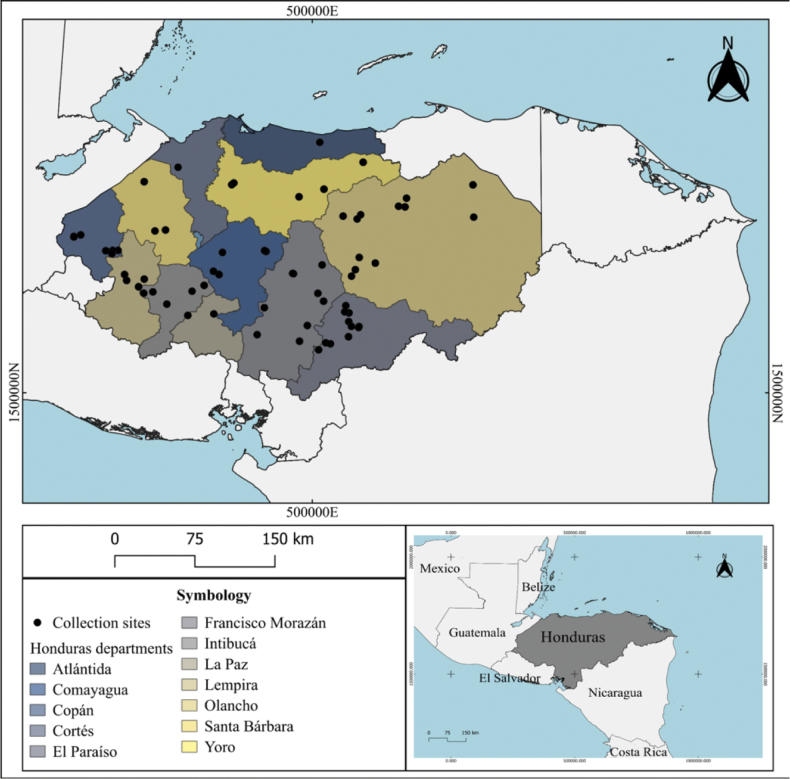
Map of Honduras showing sample collection sites in 12 departments.

During 2018–2023 a total of 1,131 insects of the order Coleoptera were collected from 12 departments of Honduras (e.g., Comayagua, Copán, Cortés, El Paraíso, Francisco Morazán, Intibucá, La Paz, Lempira, Olancho, Santa Bárbara, Yoro, and Atlántida) (Fig. 1). The data on the individuals and localities are shown in the Suppl. material 1. The specimens were trapped with Lindgren multiple funnel traps deployed consecutively throughout the entire collection time, placed approximately 0.5–1.0 m above the ground, and baited with aggregation pheromone frontalin and tree-emitted compound α-pinene. Each trap was filled with coolant liquid used for automobile radiators to preserve captured insects. In most cases, traps were inspected every two weeks to collect the specimens and transported to the “Laboratorio de Diagnóstico Sanitario Forestal” at the ICF, Tegucigalpa, Honduras. Specimens initially screened as beetles were selected, and those specimens identified as *Dendroctonus* spp. were separated from the rest of the individuals for a separate analysis whose results are not shown in this study. Consequently, this study only evaluated insect fauna considered secondary bark, ambrosia beetles, and natural predators. The selected specimens were transferred to glass vials (16 mm diameter × 50 mm height) containing 70% ethanol and transported to the Genetics Research Center at the National Autonomous University of Honduras (UNAH).

To magnify morphological features, specimens were observed under stereomicroscopes (Zeiss Stemi DV4 and UNITRON Z10 series) and identified using dichotomous keys (Wood 1982, 2007; Arnett et al. 2002; Douglas et al. 2019). Identification accuracy was assisted using bark and ambrosia beetles’ websites (https://www.barkbeetles.info/, https://ambrosiasymbiosis.org/).

The species were classified based on the feeding habits of their immature stages, as documented in the literature for each species: (a) myelophages or with feeding in the medulla; (b) phyllophagous to those that feed in the inner cortex; (c) feeding of the roots; (d) spermatophagous or seed feeding; (e) xylomycetophagous or fungal growers, and (f) xylophages to those that feed on xylem tissues. In a simplified way, all the genera that feed on phloem tissues were considered bark beetles, and those that cultivate fungi were considered ambrosial. Those that feed on tissues outside the trunk (such as seeds or roots) or internal tissues such as the pith were not considered.

### ﻿Diversity analysis

Statistical analyses were performed using the R software v. 4.3.2 (R Core Team 2023). Descriptive statistics were used to calculate the number of families, subfamilies, genera, and species collected per department. An assessment was conducted to determine the departments with the highest diversity. For this purpose, the mathematical framework of Hill numbers was used. Hill numbers differ by the parameter *q*, which determines the sensitivity of the measure to the relative abundance (Chao et al. 2014). Using the Hilldiv2 R package v. 2.0.2 (Alberdi and Gilbert 2019), we computed the orders *q* = 0 which corresponds to the species richness, and *q* = 1 which considers species according to their relative abundances and is equivalent to the Shannon’s entropy index.

### ﻿DNA extraction and barcoding

After morphological identification, individuals were stored separately in 1.5 mL tubes with 70% ethanol. Then, a small portion of soft tissue was retrieved from the abdomen for DNA barcoding analysis. A subset of 351 samples (31%) were molecularly evaluated. Those genera with less than three individuals were not sequenced since specimens were fully preserved to corroborate their identification and as vouchers. Detailed information about the sequenced samples can be found in the Suppl. material 2.

Genomic DNA was isolated using the Extracta DNA Prep for PCR kit^®^ (QuantaBio, Beverly, MA, USA) following the manufacturer’s instructions. Briefly, soft tissues were macerated and incubated at 95 °C in 100 μL of extraction reagent for 25 minutes and then cooled to room temperature for 5 minutes. Then, 100 μL of stabilization buffer was added and the final volume (200 μL) was transferred to a new 1.5 mL vial. A fragment of the mitochondrial gene COI was amplified using one of three primer pairs (Table 1).

**Table 1.** Primer sets used for amplification of the mitochondrial COI gene.

Protocol	Primer name	Sequence (5´- 3´)	Reference
A	LCO-1490	GGT CAA CAA ATC ATA AAG ATA TTG G	Folmer et al. 1994
	HCO-2198	TAA ACT TCA GGG TGA CCA AAA ATC A	–
B	BC1Fm	GTA AAA ACG ACG GCC AGT TCW AAY CAY AAR GAY ATY GG	Cho et al. 2008
	HCO-2198	–	–
C	C1-N-2650	CCN GTR AAT ARN GGG AAT CAT TG	Vink and Phillips 2007
	C1-J-2183	CAA CAT TTA TTT TGA TTT TTT GG	Simon et al. 1994

The PCR reactions were conducted in a 50 μL reaction mixture, containing 25 μL of KOD One^TM^ PCR master Mix (Toyobo Co, Ltd. Tokyo, Japan), 2 µL of each primer (100 µM), 2 µL of acetylated albumin – BSA (10 mg/mL), 15 μL of nuclease-free water, and 4 μL of DNA template. PCR reactions were performed under the following conditions: initial denaturation at 98 °C for 30 s, followed by 37 cycles of 10 s at 98 °C, annealing at 50 °C for 10 s, elongation at 68 °C for 2 min, and a final extension at 68 °C for 2 min. A negative control was included for each set of reactions. The PCR efficiency was then visualized through a 1% agarose gel electrophoresis with ethidium bromide. The resulting PCR products were purified and sequenced by Psomagen^®^ (Rockville, MD, USA) (https://www.psomagen.com).

### ﻿Bioinformatic analyses

The raw sequences were edited and assembled using Geneious^®^ prime software (Dotmatics, Boston, MA) (Kearse et al. 2012). The consensus sequences were deposited in GenBank, and accession numbers were assigned. Each sequence was subjected to a BLAST analysis on the NCBI platform (https://blast.ncbi.nlm.nih.gov/Blast.cgi) to validate the taxonomic identification. If the BLAST result revealed a percentage of identity of less than 95% concerning the result with the greatest similarity, morphology-based identification was employed to identify the deposited sequences. In addition, an open-access project titled “Project - COLEH Biodiversity of Bark beetles and other Coleoptera in Honduras” was created in the Barcode of Life Data Systems database (http://www.boldsystems.org).

To analyze the intraspecific genetic diversity of *I.apache*, 52 assembled sequences were aligned, and the resulting alignments were utilized to create a dendrogram using the Tamura-Nei genetic distance model and the Neighbor-Joining tree construction method and performing 1000 iterations of Bootstrap. A homologous sequence of *Enoclerus* sp. was included as an outgroup in the analysis. The collecting site was considered to ascertain the presence of a population structure for *I.apache*.

The number of polymorphisms among the *I.apache* sequences, the number of segregating sites (S), the average nucleotide differences (k), the number of haplotypes (H), the haplotype diversity (Hd), and the nucleotide diversity (π) were calculated using DnaSP Software (v. 6.12.03) (Rozas et al. 2017). Additionally, Tajima’s D test (Tajima 1989) was performed in DnaSP. Haplotype networks were generated using the Median Joining algorithm in Network (v. 10.2).

## ﻿Results

### ﻿Morphological identification

This study evaluated the diversity of beetles associated with coniferous woods in Honduras. Using morphological traits, we identified 27 genera of Coleoptera collected in 12 departments of Honduras. The community composition was grouped into four families and nine subfamilies (Table 2). Our analyses described three ecological groups: (a) bark beetles, (b) ambrosia beetles, and (c) natural predators, as delineated by other authors concerning the eating behavior of these genera. Most genera were classified as bark and ambrosia beetles and only about 10% as predators (Table 2). A photographic record of the genera described in this study is shown in Suppl. material 3.

**Table 2.** Identification of Coleoptera specimens at the taxonomic levels of Families, subfamilies, and genera. Mye = Myelophagy; Phl = Phylophagy (feeding on the inner cortex); RF = root feeding; Spm = spermatophagy; Xlm = xylomycetophagy; Xyl = xylophagy; NA = not applicable; ND = not defined.

N	Family	Subfamily	Genus	Ecological group (Reference)	Category	Number (%)
1	Bostrichidae	Dinoderinae	*Stephanopachys*	Xyl (Covre et al. 2023)	NA	1 (0.09%)
2		Bostrichinae	*Xylomeira*	Xyl (Covre et al. 2023)	NA	1 (0.09%)
3	Curculionidae	Cossoninae	*Tomolips*	ND (Jordal 2014)	NA	61 (5%)
4			*Stenoscelis*	ND (Jordal 2014)	NA	2 (0.2%)
5		Entiminae	*Mimographus*	RF (Lanteri et al. 2002)	NA	1 (0.09%)
6		Platypodinae	*Euplatypus*	Xlm (Kirkendall et al. 2015)	Ambrosia	35 (3%)
7			*Tesserocerus*	Xlm (Kirkendall et al. 2015)	Ambrosia	1 (0.09%)
8		Scolytinae	*Araptus*	Phl, Spm (Kirkendall et al. 2015)	Bark	1 (0.09%)
9			*Cryptocarenus*	Spm (Kirkendall et al. 2015)	NA	10 (0.9%)
10			*Coccotrypes*	Phl, Spm (Kirkendall et al. 2015)	Bark	1 (0.09%)
11			*Corthylus*	Xlm (Kirkendall et al. 2015)	Ambrosia	2 (0.2%)
12			*Dendroctonus*	Phl (Kirkendall et al. 2015)	Bark	4 (0.3%)
13			*Gnathotrichus*	Xlm (Kirkendall et al. 2015)	Ambrosia	22 (2%)
14			*Hylastes*	Phl (Kirkendall et al. 2015)	Bark	6 (0.5%)
15			*Hylocurus*	Phl (Kirkendall et al. 2015)	Bark	1 (0.09%)
16			*Hypothenemus*	Phl, Spm, Xlm (Kirkendall et al. 2015)	Bark/Ambrosia	24 (2%)
17			*Ips*	Phl (Kirkendall et al. 2015)	Bark	602 (53%)
18			*Monarthrum*	Xlm (Kirkendall et al. 2015)	Ambrosia	3 (0.3%)
19			*Micracis*	Xyl (Kirkendall et al. 2015)	NA	1 (0.09%)
20			*Pityophthorus*	Mye, Phl	Bark	16 (1.4%)
21			*Taurodemus*	Xlm (Kirkendall et al. 2015)	Ambrosia	1 (0.09%)
22			*Xyleborinus*	Xlm (Kirkendall et al. 2015)	Ambrosia	14 (1.2%)
23			*Xyleborus*	Xlm (Kirkendall et al. 2015)	Ambrosia	187 (16.5%)
24	Cleridae	Clerinae	*Enoclerus*	Pred (Wegensteiner et al. 2015)	Predator	21 (1.8%)
25		Tilinae	*Cymatodera*	Pred (Wegensteiner et al. 2015)	Predator	1 (0.09%)
26	Trogossitidae	Trogossitinae	*Temnoscheila*	Pred (Wegensteiner et al. 2015)	Predator	111 (10%)
27			*Tenebroides*	Pred (Wegensteiner et al. 2015)	Predator	1 (0.09%)
**Total**						**1131 (100%)**

In terms of relative abundance, our results revealed that *Ips* (54%), *Xyleborus* (16.5%), *Temnoscheila* (10%), *Tomolips* (5%), *Euplatypus* (3%), *Hypothenemus* (2%), and *Gnathotrichus* (2%) comprised more than 93% of the total collected individuals, whereas the other 20 genera accounted 7% of the collection. In addition, according to the number of elytral declivity spines, two species of *Ips* were identified: *I.apache and I.cribricollis*, most of them collected in Francisco Morazán, Yoro, and Olancho.

**Figure 2.**
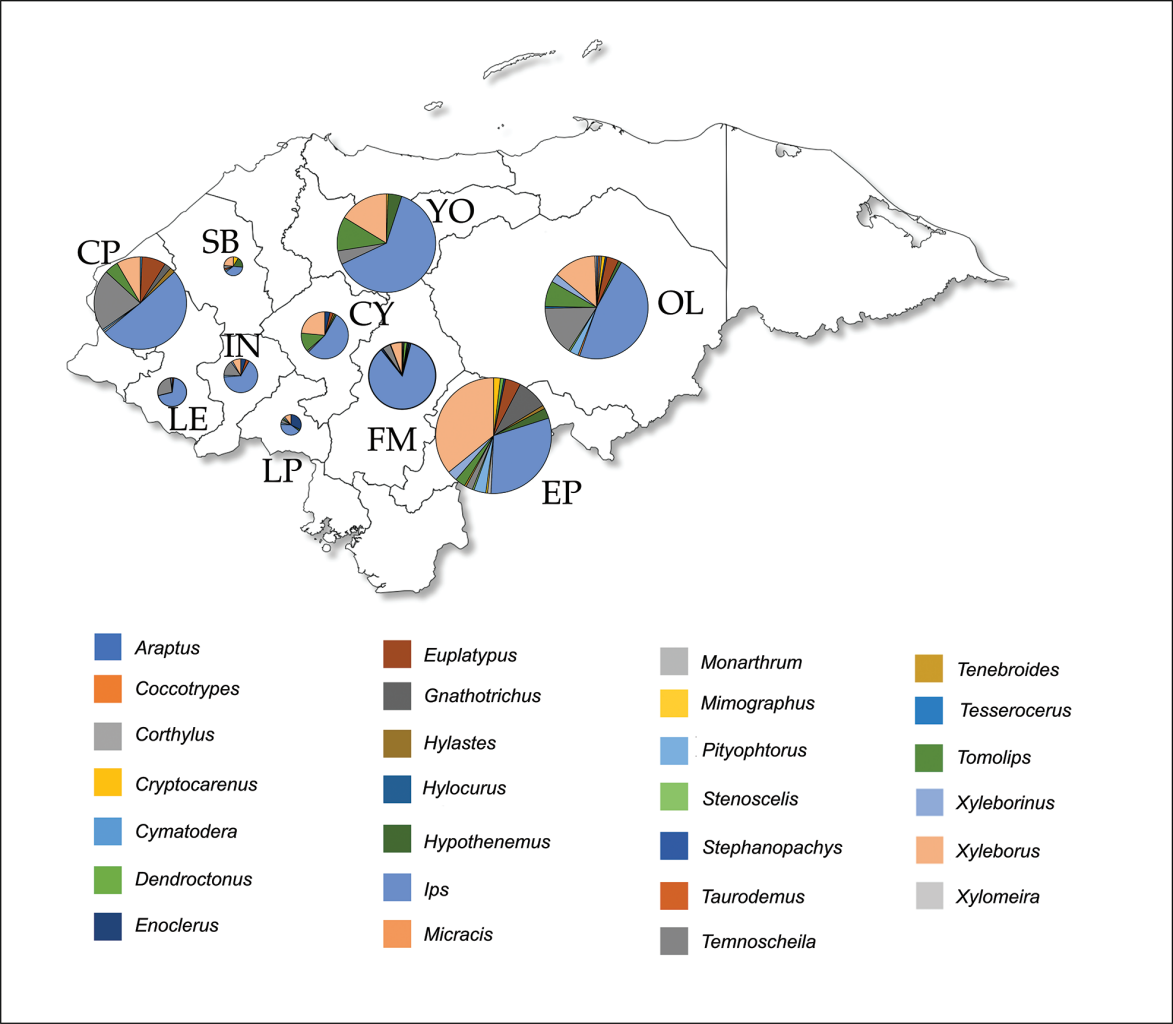
Map of Honduras showing the proportion of genera identified in eleven departments of Honduras. The size of the pie diagrams is proportional to the number of insects identified compared to the total. In the department of Cortés, a single individual of the genus *Temnoscheila* was identified, which is not shown in the graph. Department codes: CP: Copán; CY: Comayagua; EP: El Paraíso; FM: Francisco Morazán; IN: Intibucá; LP: La Paz; LE: Lempira; OL: Olancho; SB: Santa Bárbara; YO: Yoro.

The largest number of specimens of Coleoptera were collected in El Paraíso (*n* = 209; 18.5%), followed by Olancho (*n* = 186; 16.4%), Yoro (*n* = 178; 15.7%), Copán (*n* = 167; 14.8%), Francisco Morazán (*n* = 121; 10.7%). The remaining departments contributed less than 7% of the total (Fig. 2). To our knowledge, this is the first report of the presence of *Xylomeira* and *Stephanopachys* in Honduran pine forests.

### ﻿Diversity analysis

**Figure 3.**
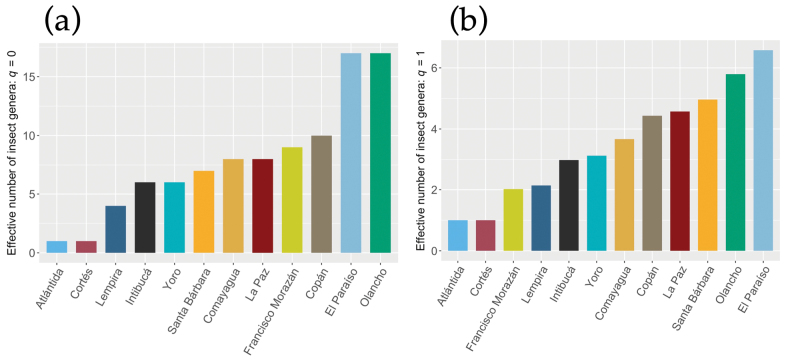
Bar plots depicting the effective number of genera at orders *q* = 0 (a) and *q* = 1; (b) among the 12 departments.

Diversity of specimens was computed using the Hill numbers by parameterizing the *q* value. For instance, diversity analysis at order *q* = 0 revealed that four departments have the greatest richness of genera: Olancho and El Paraíso have effective numbers of 17, Copá*n* = 10, and Francisco Morazá*n* = 9; whereas taxonomic diversity at order *q* = 1 increased in El Paraíso (*n* = 6.5), Olancho (*n* = 5.8), Santa Bárbara (*n* = 5), La Paz (*n* = 4.5) and Copán (*n* = 4.4) (Fig. 3). While these departments displayed greater diversity, the low diversity for the remaining sites could be related to the small sample sizes and therefore affected the statistical power of our analysis.

### ﻿Barcoding

DNA was isolated from 376 individuals, and successful amplification was achieved for 212 (56.4%) using at least one of the PCR procedures, from which 124 sequences (55.5%) yielded a high-quality score. A total of 56 sequences were identified as *I.apache* (44.8%), 21 as *I.cribricollis* (16.8%), 12 as *Xyleborus* spp. (10.4%), 6 as *Euplatypus* spp. (4.8%), and 5 as *Temnoscheila* spp. (4.0%). Additionally, five sequences were from *Gnathotrichus* spp. and *Hypothenemus* spp., and three sequences were from *Cryptocarenuslepidus*. Moreover, four sequences were from *Enoclerus* spp., two sequences each from *Pityophthorus* spp. and *Tomolips* spp., and one sequence each from *Hylastes* spp. and *Xyleborinus* spp. GenBank accession numbers and BOLD identification numbers (BINs) are listed in Suppl. material 2. Most of the sequences obtained in this study are the first reported in GenBank for the identified species. Furthermore, one of the sequences was identified as a mite of the taxon *Trichouropoda*, with 85% percent identity in NCBI. Three sequences were identified as the intracellular bacterium *Rickettsiabelli* with 97% identity. Moreover, six sequences were identified as the nematode *Deladenus* spp. with identity percentages between 86–89%.

### ﻿Intraspecific diversity of *Ipsapache*

**Figure 4.**
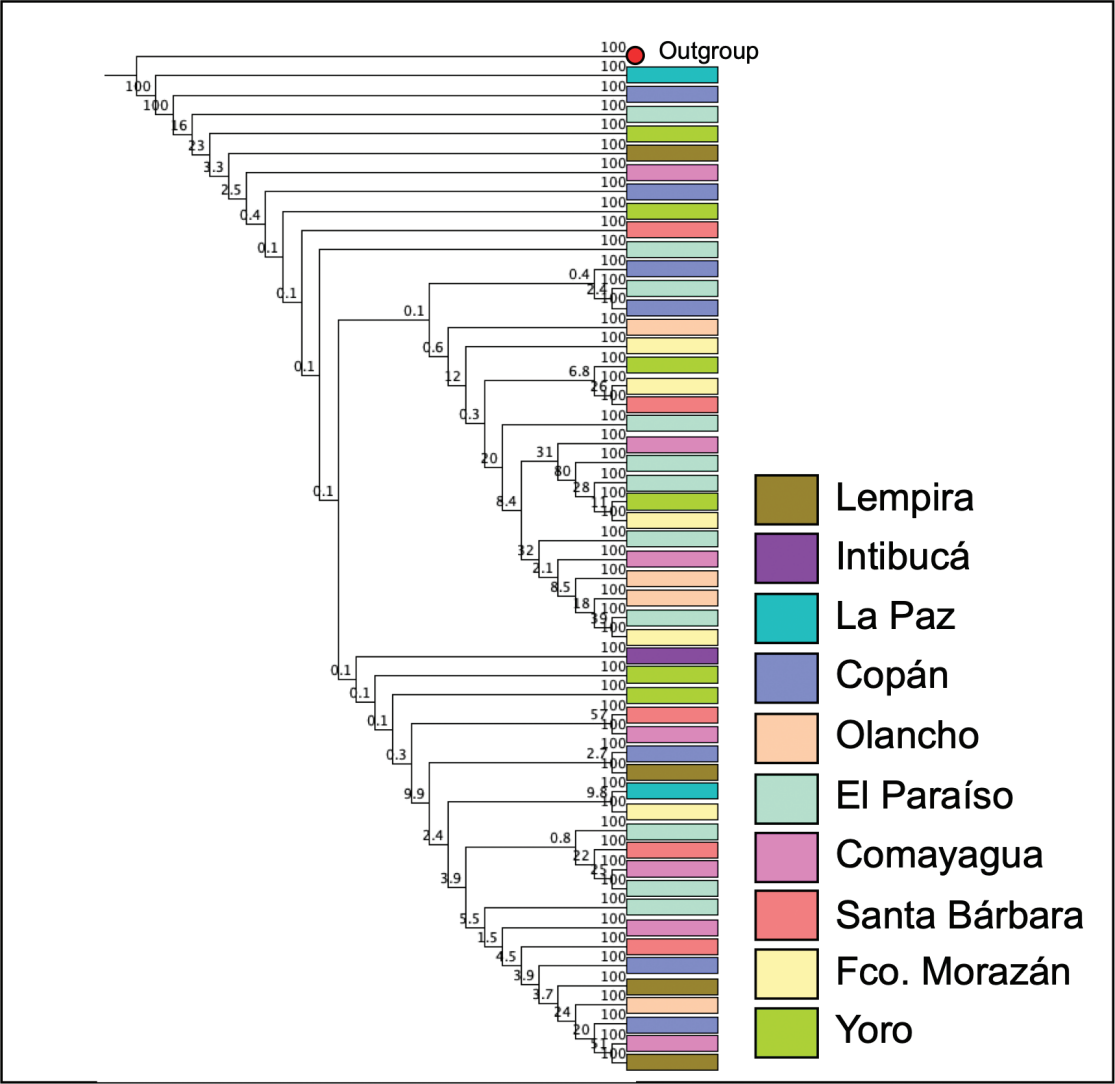
Dendrogram of *Ipsapache*. The colored boxes indicate the department in which the specimens were collected. The red dot indicates the *Enoclerus* sequence used as an outgroup.

Regarding the analysis of intraspecific diversity of *I.apache*, it was observed that among 51 sequences with a length of up to 630 bp, the number of identical sites was 568 (81.5%), and the Pairwise % identity coefficient was 98.6%. A population structure related to the collection site was not demonstrated for *I.apache* (Fig. 4).

**Figure 5.**
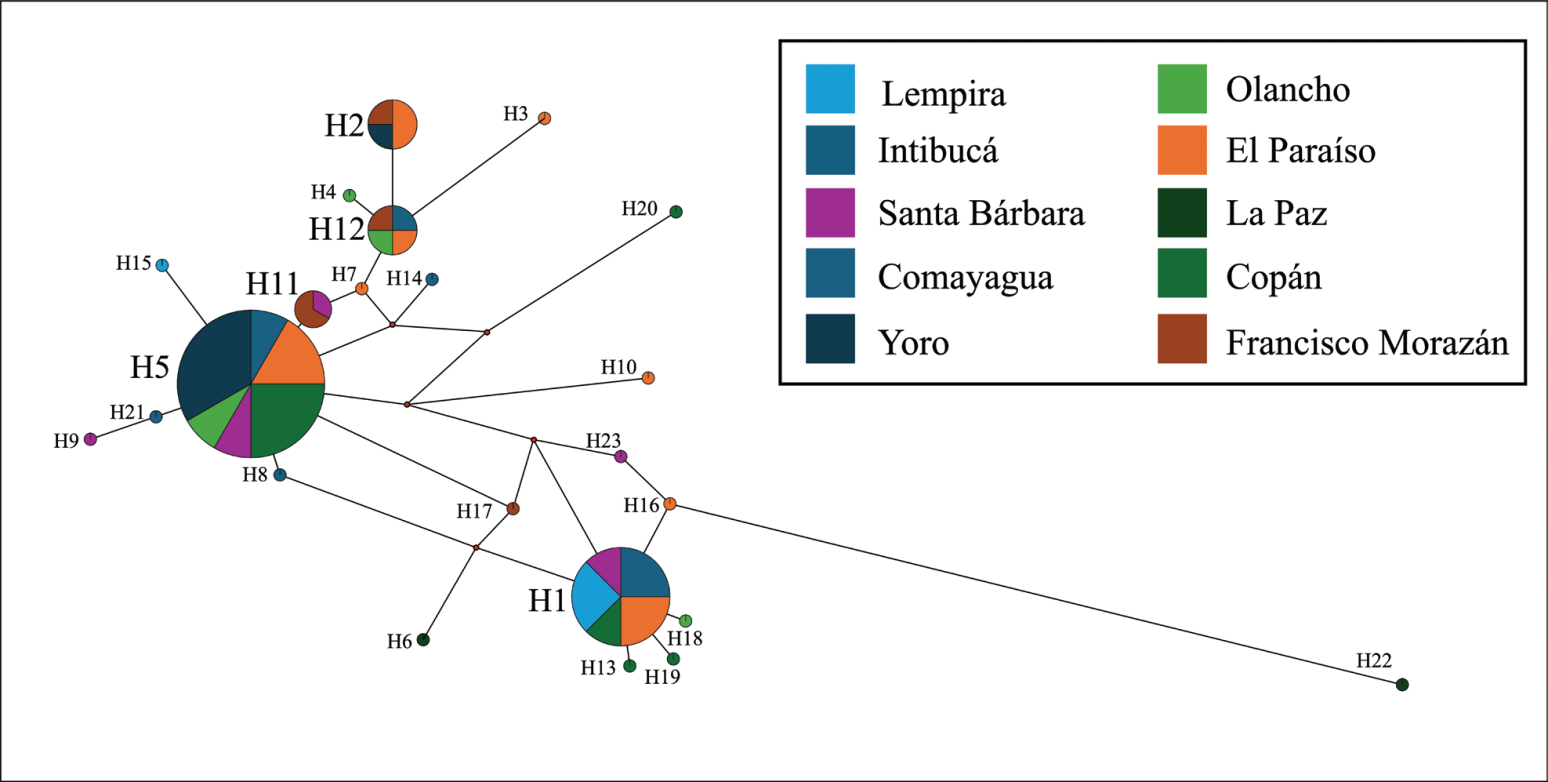
Haplotype network of 49 *Ipsapache* sequences according to the specimen collection department.

To analyze the number of haplotypes in the *I.apache* population, 49 sequences of 527 bp were aligned. The number of segregating sites (S) was 93. The nucleotide diversity (π) was equal to 0.0142, and the average nucleotide difference (k) was 7.156. Twenty-three haplotypes (H) were found (Fig. 5), with a diversity index (Hd) of 0.9073 (SD = 0.027). Haplotypes 5 and 1 were the most frequent, with 12 and 8 sequences respectively. 18 of 23 (78.3%) haplotypes included a single sequence. Haplotype 11 was formed with 3 sequences, while haplotypes 2 and 12 were formed with 4 sequences each. The haplotype network was constructed considering the department of origin of the specimen (Fig. 5). No type of correlation was observed between the haplotypes and the collection site.

To explore the influence of natural selection on population structure, Tajima’s D test was performed on the COI sequences and calculated a D value of –2.45039 (Statistical significance: **, *p* < 0.02). Fu and Li’s D* and F* tests resulted in values of –5.47644 and –5.19970 respectively (Statistical significance: **, *p* < 0.02), suggesting an excess of rare variation, consistent with population growth, or positive selection.

## ﻿Discussion

Limited research has been conducted on the biodiversity of beetle fauna associated with coniferous forests in Honduras, restricting our understanding of their ecological and functional roles. Therefore, a better understanding of insect inventories and spatial distribution is required to increase awareness of beetle outbreaks and their negative impacts on ecosystem services at regional scales. In this study, we used a combination of morphological and molecular methods to analyze the community composition of bark and ambrosia beetles, along with their natural predators, in pine forests. Overall, our integrative analysis provided a more complete and suitable assessment of the forest insect community at lower taxonomic levels (e.g. genus and species). In line with our findings, recent studies have also evidenced the suitability of integrated morphological and molecular approaches to taxonomic analysis in insect communities (Landi et al. 2017; Tahir et al. 2018; Wu et al. 2021; Zhang et al. 2022).

### ﻿Beetle diversity and spatial distribution

Our collection revealed a high diversity of beetles, composed of 27 genera, with the majority found in the subfamily Scolytinae (60%). *Ips*, *Temnoscheila*, and *Xyleborus* displayed the broadest range of distribution in Honduras. Recent studies have suggested that these beetles play a crucial role in forest ecosystem dynamics, enabling ecological balance, natural renewal process, forest structure, and succession (Andrei and Ifrim 2021; Gandhi and Hofstetter 2021). In addition, the large-scale geographical distribution and colonization of these genera can be related to their high dispersal capacity over short distances within a forest stand or long distances above the forest canopy (Sallé et al. 2007; Jones et al. 2019). Particularly, it has been argued that *Ips* spp. can fly above the canopy and move up to 55 km for several hours (Jones et al. 2019), and thus can colonize coniferous forests over long distances. The extensive distribution of *Ips* spp. (secondary pest), in addition to the previously reported prevalence of the southern pine beetle *Dendroctonusfrontalis* (primary pest) (Rivera-Rojas et al. 2010; Hernandez et al. 2012) is particularly noteworthy due to the potential resource conflict that may occur between them (Smith et al. 2021). However, further studies are needed to properly address their interspecific competition at temporal and spatial scales.

According to the statistical analysis at the genus level, Olancho and El Paraíso harbored the greatest beetle richness (*q* = 0). However, the relative abundance of common genera (*q* = 2) increased in El Paraíso compared to Olancho, suggesting a low presence of rare beetles with an increasing number of common genera in the community. It has been mentioned that rare beetle species are more sensitive to environmental changes (Haack et al. 2022), thereby their low presence in El Paraíso could be partially explained by the greatest attacks by bark beetles (*Dendroctonus* spp. and *Ips* spp.) in the last decades, losing at least 265 ha of healthy forest. The long-term consequences of bark beetle attacks have resulted in extensive tree mortality, alteration of forest structure, and low productivity. Despite this continuous and persistent infestation, the high degree of ecological integrity and resilience of pine forests seems to maintain a great diversity of beetles.

Our findings revealed that the secondary bark beetle species of the genus *Ips* and their natural enemies, i.e., *Temnoscheila* (Trogossitidae) and *Enoclerus* (Cleridae), occur simultaneously in most sampled sites, indicating an overlapping distribution. Both genera have been extensively identified as associated predators of harmful forest pests such as *Ips* and *Dendroctonus* species (Wegensteiner et al. 2015). While *Temnoscheila* and *Enoclerus* showed a greater diversity and broad distribution (Kolibáč 2013; Rifkind 2021), their diversity, natural history traits, population dynamics, and interaction with primary/secondary bark beetles have been little explored in Honduras. Additionally, the results showed a positive response of bark beetles and their associated predators to Lindgren funnel traps baited with frontalin and α-pinene. The use of targeted semiochemicals in Scolytinae trapping studies provides a unique opportunity to assess their associated insects and improve biological control strategies. For instance, Aukema & Raffa (Aukema and Raffa 2005) reported that *Thanasimusdubius* (Cleridae) was strongly attracted to frontalin and α-pinene pheromones. However, further research on selective attraction as well as testing new combinations of semiochemicals will improve forest management strategies during bark beetle infestation.

### ﻿Genetic diversity and neighbor-joining analysis

DNA barcoding has become a powerful tool for estimating intraspecific genetic diversity in Coleoptera (Jordal and Kambestad 2014; Kiran et al. 2019; Ho et al. 2022; Kabalak et al. 2022). However, the effectiveness of DNA barcoding depends on comprehensive reference libraries. If the reference database lacks sequences for certain species, accurate identification becomes challenging. For most of the genera and species described in this study, no sequences of the same COI gene segment were available in existing databases. Consequently, it was not possible to compare these sequences with those obtained from specimens collected in other countries. Therefore, this study presents, for the first time, partial sequences of the COI gene from many different species and genera of beetles that reside in coniferous forests in Honduras.

On the other hand, the neighbor-joining analysis and the haplotype network show intraspecific genetic differences among individuals of *I.apache*, however the sequences clustered without any clear structure. Moreover, a π value of 0.0142 suggests a moderate level of genetic diversity whilst a haplotype diversity (Hd) of 0.9073, indicates a high level of genetic variability. A lack of genetic differentiation among populations coupled with moderate to high levels of genetic diversity, may suggest gene flow among individuals inhabiting different coniferous forests in Honduras. In addition, the predominance of two haplotypes implies that certain haplotypes are more common within the population, which could be due to selective advantages or historical demographic events.

Additionally, neutrality tests indicate an excess of rare genetic variation, consistent with either population growth or positive selection. This suggests that *I.apache* populations are likely well-adapted to the environmental conditions of Honduran coniferous forests, enhancing their persistence and facilitating their spread within these habitats. *Ipsapache* is a well-known species for its host specialization and feeding primarily on pine forests (Cognato 2015). Hence, a level of substantial genetic diversity and allelic richness, coupled with limited population differentiation, might be attributed in part to the lack of physical barriers and favorable environmental conditions that promote population expansion. In addition, given that *Ips* bark beetles are a priority issue for local governments, predicting future trajectories will provide a solid scientific basis to estimate their impacts on forest ecosystems. However, more genetic and ecological studies are needed to assess the genetic divergence and environmental adaptations of *Ips* engraver beetles in Honduran coniferous forests

## ﻿Conclusions

As far as we know, this research is one of the first investigations conducted on the diversity of beetles associated with coniferous forests in Honduras. A total of 1,131 individuals were examined, revealing 27 genera, with *Ips* being the most prevalent, followed by *Xyleborus* and *Temnoscheila*. Species-level identification was achieved for 68% of the specimens, while all were identified to the genus level. *Ips*, *Temnoscheila*, *Xyleborus*, *Hypothenemus*, and *Pityophthorus* exhibited the broadest geographic distribution across the sampled sites. Olancho, El Paraíso, and Copán demonstrated the greatest genus-level diversity. Additionally, this study presents the first record of the genera *Xylomeira* and *Stephanopachys* in Honduran pine forests. Furthermore, the mitochondrial COI gene reveals significant genetic diversity among *I.apache* populations but no structure, suggesting gene flow among individuals from different localities. Additionally, due to the greater impact of bark beetles in Honduran pine forests, our findings are important for pest management strategies and phytosanitary measures to prevent the risk of introduction and spread of harmful beetle species. Lastly, future studies should focus on understanding the trophic relationships between bark beetles and natural predators, providing valuable insights into fundamental ecological processes in coniferous forests.

## ﻿Additional information

### Author ORCIDs

Mauricio Hernández https://orcid.org/0000-0003-0205-3344

Mauricio Michel https://orcid.org/0009-0009-2331-6810

Joel García https://orcid.org/0000-0002-0574-758X

Marcela Moncada https://orcid.org/0009-0003-2380-1548

Alejandra Pinto https://orcid.org/0000-0003-3890-0218

Gabriela Matamoros https://orcid.org/0000-0003-2177-9765

Gustavo Fontecha https://orcid.org/0000-0001-9756-4520

### Data availability

All of the data that support the findings of this study are available in the main text or Supplementary Information.

## Supplementary materials

Supplementary material 1 Database with the identification of the specimens in the collection Data type xlsx https://binary.pensoft.net/file/1243712This dataset is made available under the Open Database License (http://opendatacommons.org/licenses/odbl/1.0/). The Open Database License (ODbL) is a license agreement intended to allow users to freely share, modify, and use this Dataset while maintaining this same freedom for others, provided that the original source and author(s) are credited.Mauricio Hernández, Mauricio Michel, Joel García, Geisy Dueñas, Marcela Moncada, Kevin Amaya, Yensi Yánez, Alejandra Pinto, Gabriela Matamoros, Alejandro Zamora, Gustavo Fontecha

Supplementary material 2 GenBank accession numbers and BOLD BINs for the sequences generated in this study Data type xlsx https://binary.pensoft.net/file/1243713This dataset is made available under the Open Database License (http://opendatacommons.org/licenses/odbl/1.0/). The Open Database License (ODbL) is a license agreement intended to allow users to freely share, modify, and use this Dataset while maintaining this same freedom for others, provided that the original source and author(s) are credited.Mauricio Hernández, Mauricio Michel, Joel García, Geisy Dueñas, Marcela Moncada, Kevin Amaya, Yensi Yánez, Alejandra Pinto, Gabriela Matamoros, Alejandro Zamora, Gustavo Fontecha

Supplementary material 3 Photographic catalog of the genera of Coleoptera described in this study Data type docx Explanation note Species are listed in order of abundance. https://binary.pensoft.net/file/1243714This dataset is made available under the Open Database License (http://opendatacommons.org/licenses/odbl/1.0/). The Open Database License (ODbL) is a license agreement intended to allow users to freely share, modify, and use this Dataset while maintaining this same freedom for others, provided that the original source and author(s) are credited.Mauricio Hernández, Mauricio Michel, Joel García, Geisy Dueñas, Marcela Moncada, Kevin Amaya, Yensi Yánez, Alejandra Pinto, Gabriela Matamoros, Alejandro Zamora, Gustavo Fontecha
