# Supplementary material for: ﻿Diversity of beetles (Arthropoda, Insecta, Coleoptera) associated with coniferous forests in Honduras
Source: Zookeys. 2025 Feb 6;1226:101–19. doi: 10.3897/zookeys.1226.136987 (PMC11826226; doi:10.3897/zookeys.1226.136987)
Supplement: Supplementary material 3 — Photographic catalog of the genera of Coleoptera described in this study [file zookeys-1226-101_article-136987__-s003.docx]

## Photo annex

| **N** | **Taxonomy** | **Dorsal view** | **Lateral view** |
| --- | --- | --- | --- |
| 1 | Class: Insecta  Order: Coleoptera  Family: Curculionidae  Subfamily: Scolytinae  Genus: *Ips*  Species: *apache* | **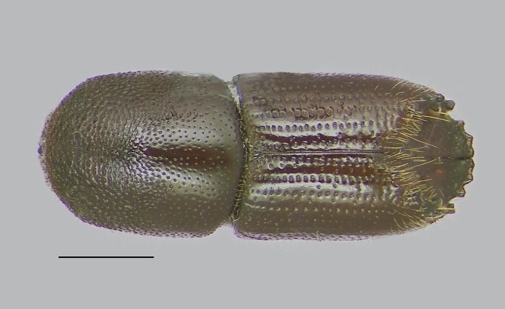** | **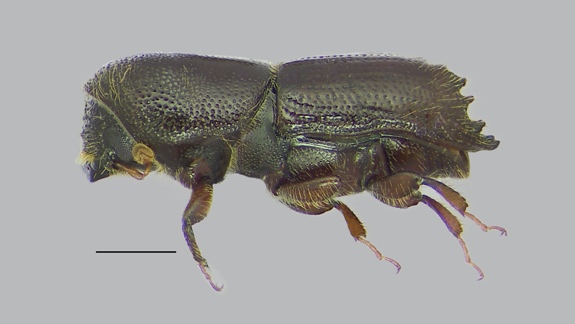** |
| 2 | Class: Insecta  Order: Coleoptera  Family: Curculionidae  Subfamily: Scolytinae  Genus: *Ips*  Especie: *cribricollis* | **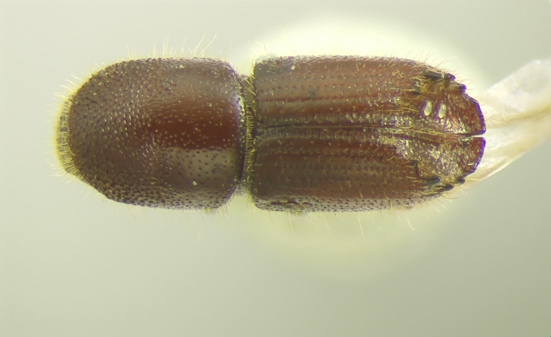** | **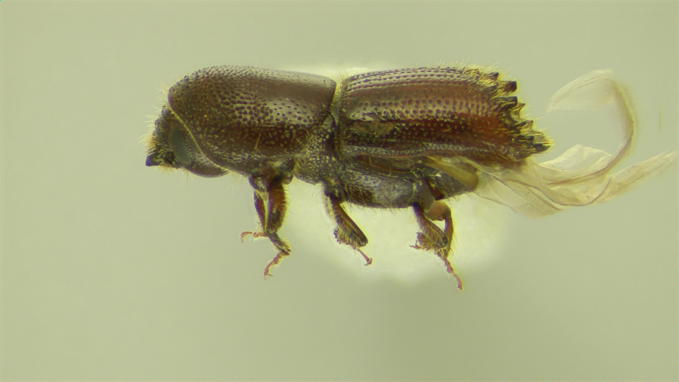** |
| 3 | Class: Insecta  Order: Coleoptera  Family: Curculionidae  Subfamily: Scolytinae  Genus: *Xyleborus* | **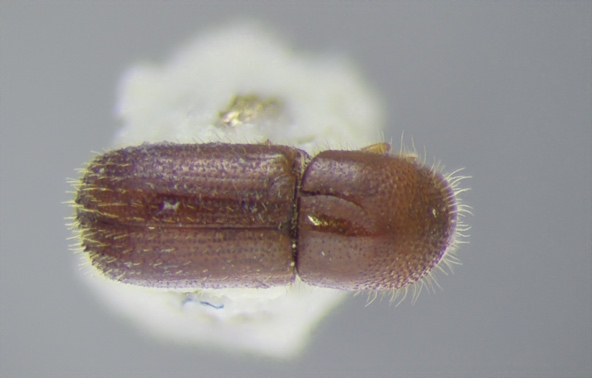** | **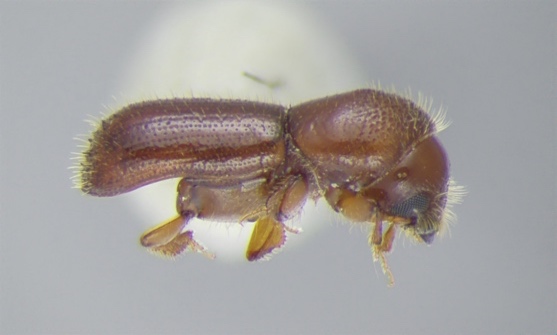** |
| 4 | Class: Insecta  Order: Coleoptera  Family: Trogossitidae  Subfamily: Trogossitinae  Genus: *Temnoscheila* | **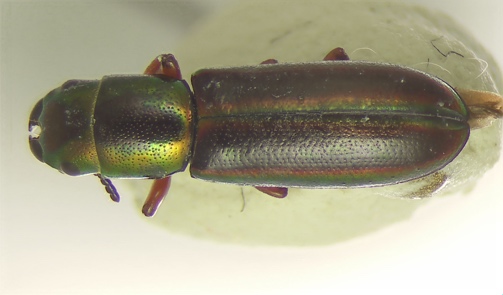** | **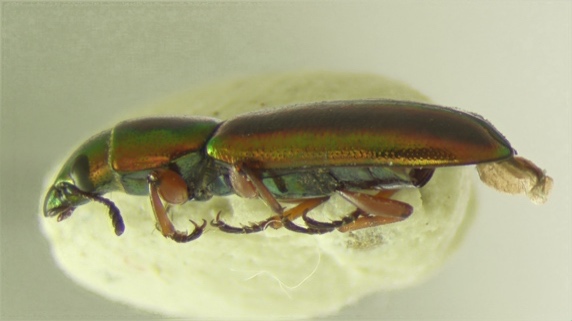** |
| 5 | Class: Insecta  Order: Coleoptera  Family: Curculionidae  Subfamily: Cossoninae  Genus: *Tomolips* | **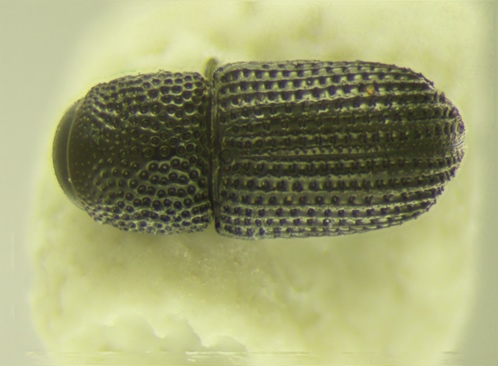** | **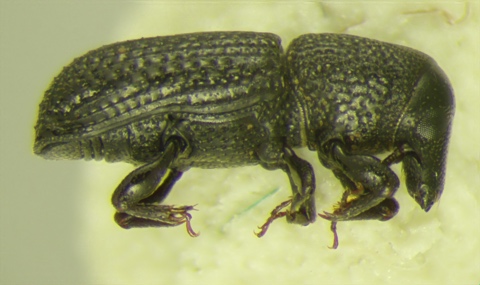** |
| 6 | Class: Insecta  Order: Coleoptera  Family: Curculionidae  Subfamily: Platypodinae  Genus: *Euplatypus* | **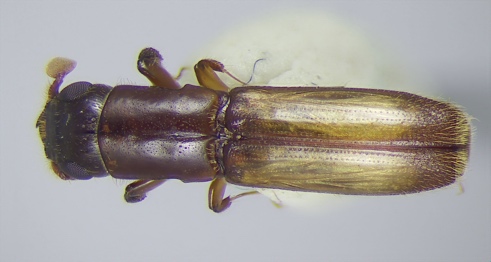** | **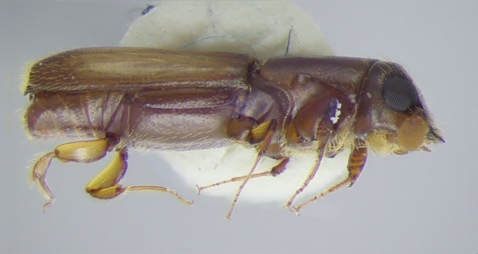** |
| 7 | Class: Insecta  Order: Coleoptera  Family: Curculionidae  Subfamily: Scolytinae  Genus: *Hypothenemus* | **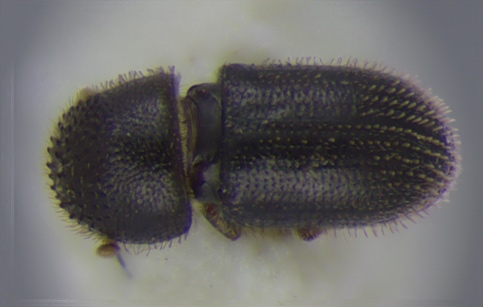** | **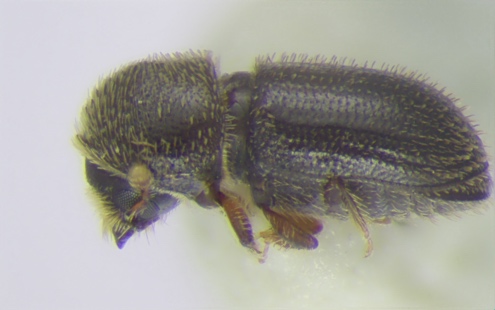** |
| 8 | Class: Insecta  Order: Coleoptera  Family: Curculionidae  Subfamily: Scolytinae  Genus: *Gnathotrichus* | **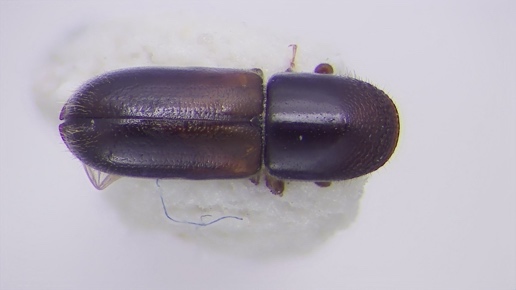** | **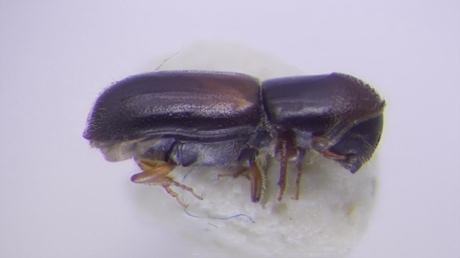** |
| 9 | Class: Insecta  Order: Coleoptera  Family: Cleridae  Subfamily: Clerinae  Genus: *Enoclerus* | **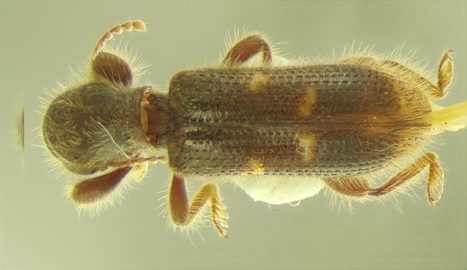** | **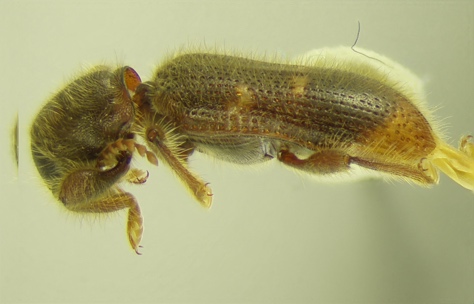** |
| 10 | Class: Insecta  Order: Coleoptera  Family: Curculionidae  Subfamily: Scolytinae  Genus: *Pityophthorus* | **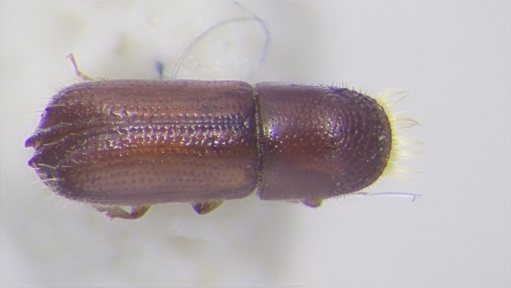** | **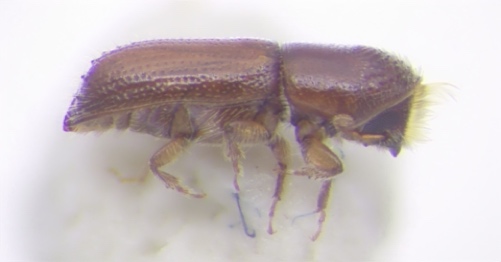** |
| 11 | Class: Insecta  Order: Coleoptera  Family: Curculionidae  Subfamily: Scolytinae  Genus: *Xyleborinus* | **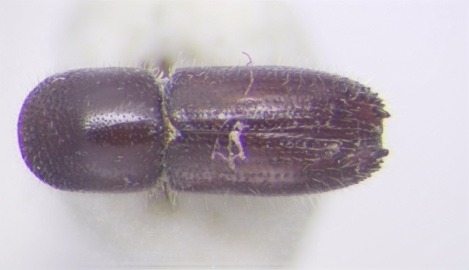** | **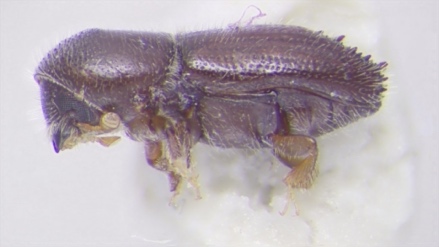** |
| 12 | Class: Insecta  Order: Coleoptera  Family: Curculionidae Subfamily: Scolytinae  Genus: *Cryptocarenus* | **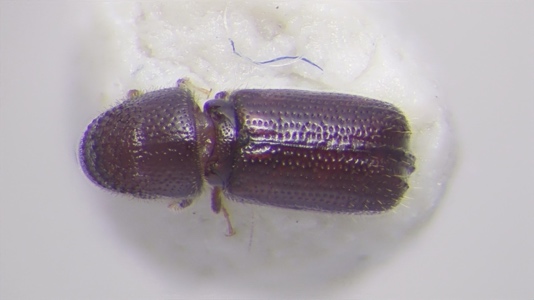** | **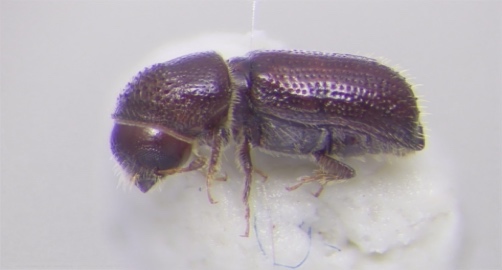** |
| 13 | Class: Insecta  Order: Coleoptera  Family: Curculionidae  Subfamily: Scolytinae  Genus: *Hylastes* | **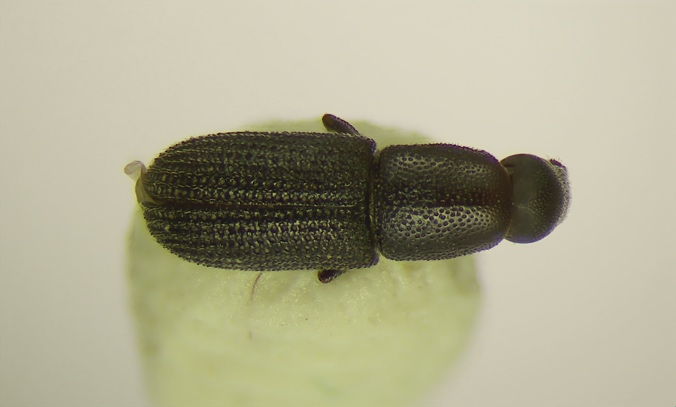** | **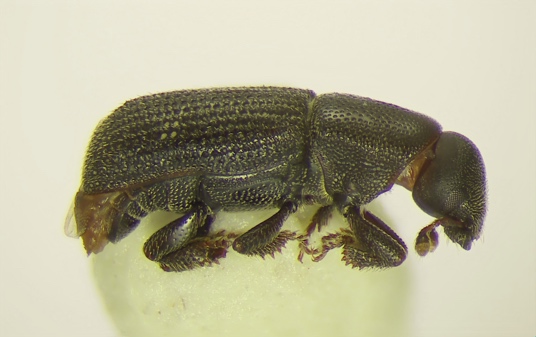** |
| 14 | Class: Insecta  Order: Coleoptera  Family: Curculionidae  Subfamily: Scolytinae  Genus: *Dendroctonus* | **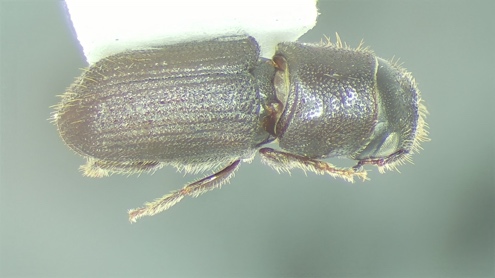** | **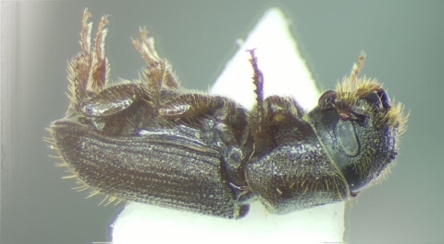** |
| 15 | Class: Insecta  Order: Coleoptera  Family: Curculionidae  Subfamily: Scolytinae  Genus: *Monarthrum* | **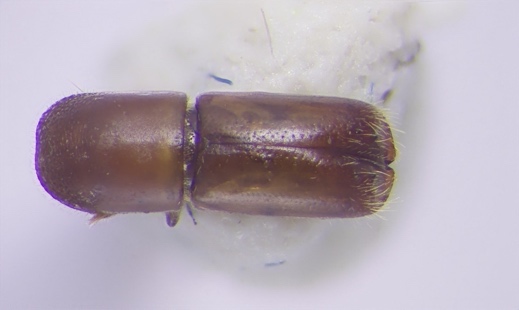** | **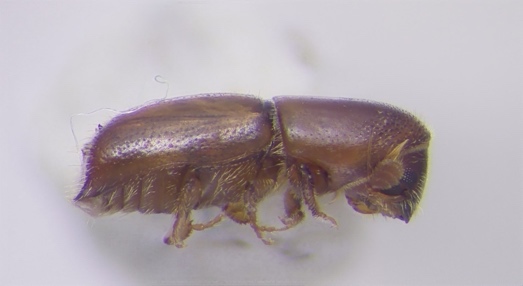** |
| 16 | Class: Insecta  Order: Coleoptera  Family: Curculionidae Subfamily: Scolytinae  Genus: *Corthylus* | **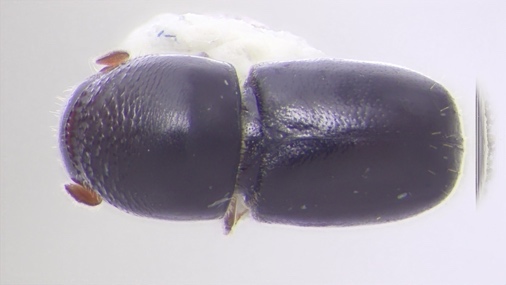** | **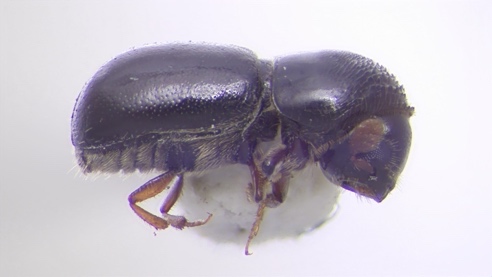** |
| 17 | Class: Insecta  Order: Coleoptera  Family: Curculionidae  Subfamily: Cossoninae  Genus: *Stenoscelis* | **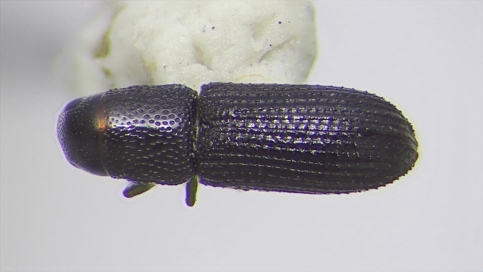** | **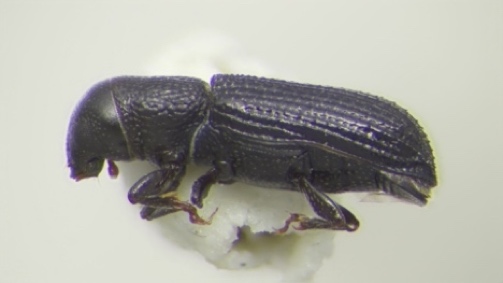** |
| 18 | Class: Insecta  Order: Coleoptera  Family: Curculionidae  Subfamily: Scolytinae  Genus: *Araptus* | **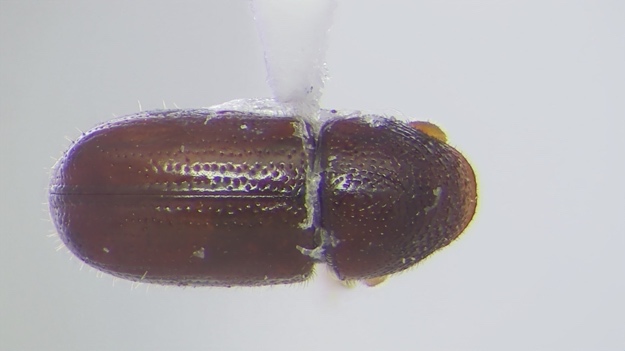** | **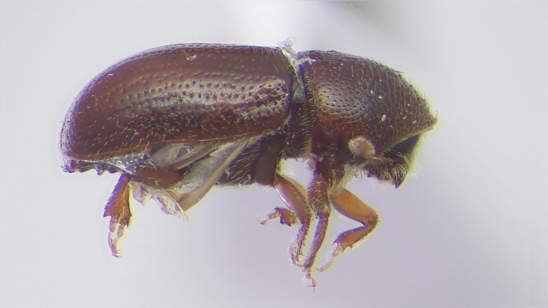** |
| 19 | Class: Insecta  Order: Coleoptera  Family: Curculionidae  Subfamily: Scolytinae  Genus: *Coccotrypes* | **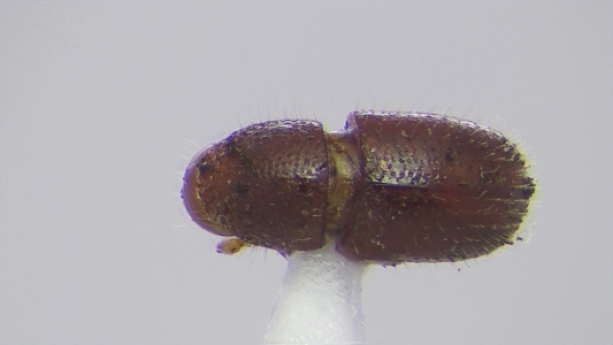** | **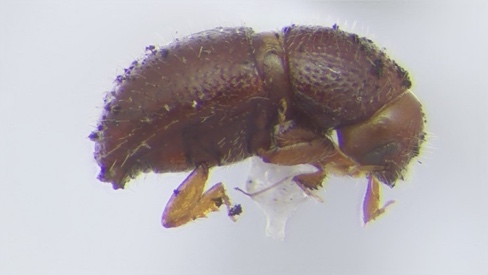** |
| 20 | Class: Insecta  Order: Coleoptera  Family: Cleridae  Subfamily: Tilinae  Genus: *Cymatodera* | **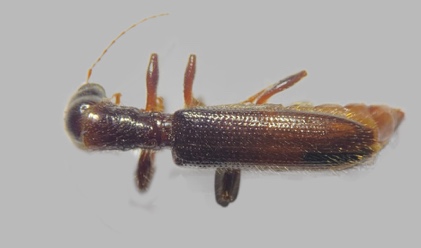** | **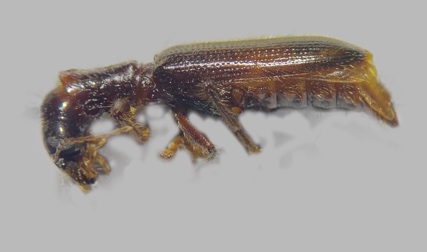** |
| 21 | Class: Insecta  Order: Coleoptera  Family: Curculionidae  Subfamily: Scolytinae  Genus: *Hylocurus* | **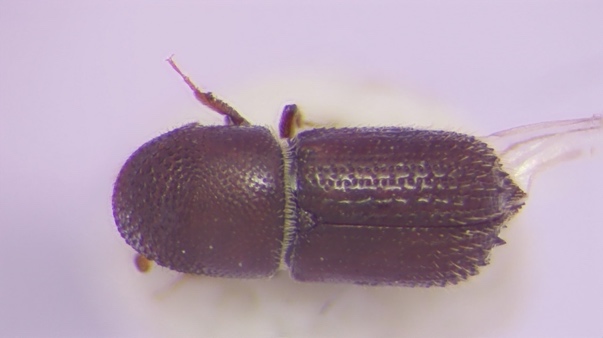** | **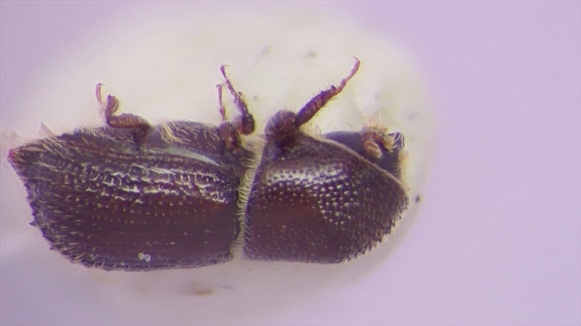** |
| 22 | Class: Insecta  Order: Coleoptera  Family: Curculionidae  Subfamily: Scolytinae  Genus: *Micracis* | **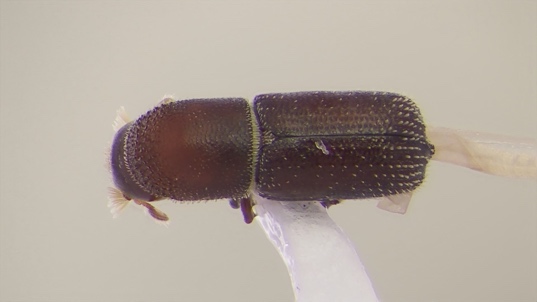** | **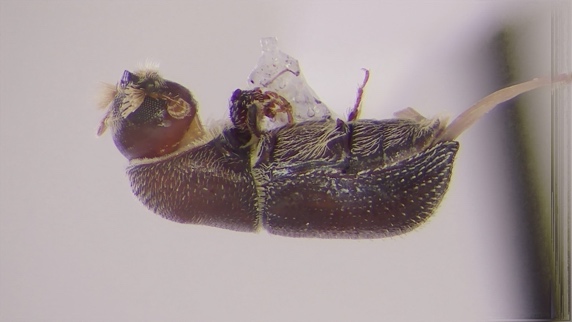** |
| 23 | Class: Insecta  Order: Coleoptera  Family: Curculionidae  Subfamily: Entiminae  Genus: *Pantomorus* | **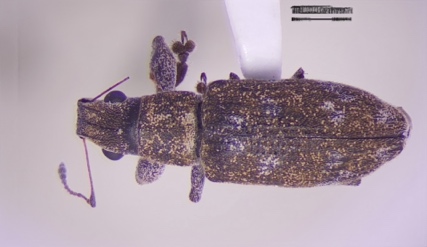** | **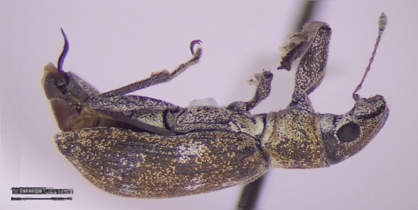** |
| 24 | Class: Insecta  Order: Coleoptera  Family: Bostrichidae  Subfamily: Dinoderinae  Genus: *Stephanopachys* | **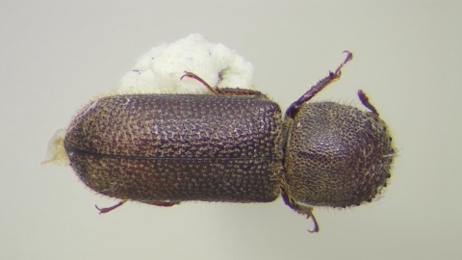** | **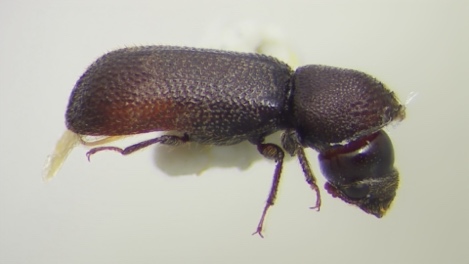** |
| 25 | Class: Insecta  Order: Coleoptera  Family: Curculionidae  Subfamily: Scolytinae  Genus: *Taurodemus* | **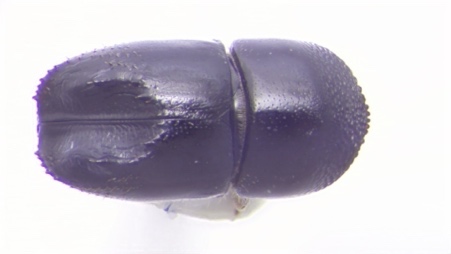** | **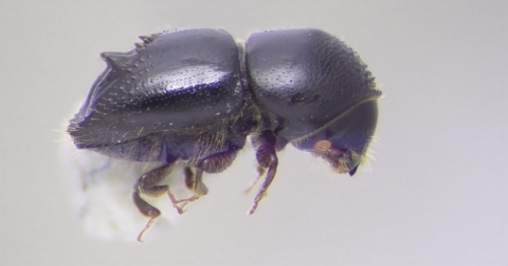** |
| 26 | Class: Insecta  Order: Coleoptera  Family: Curculionidae  Subfamily: Scolytinae  Genus: *Tenebroides* | **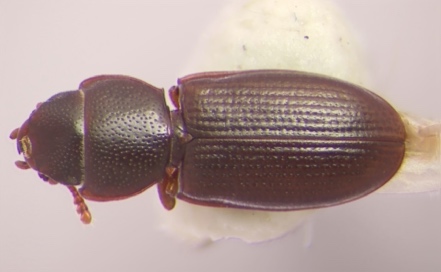** | **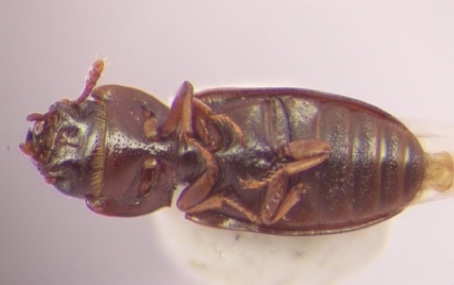** |
| 27 | Class: Insecta  Order: Coleoptera  Family: Curculionidae  Subfamily: Platypodinae  Genus: *Tesserocerus* | **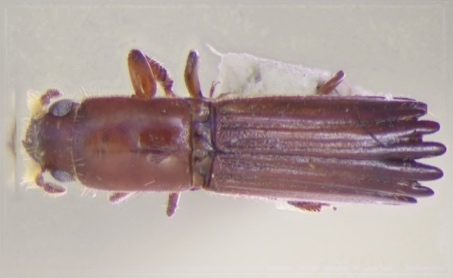** | **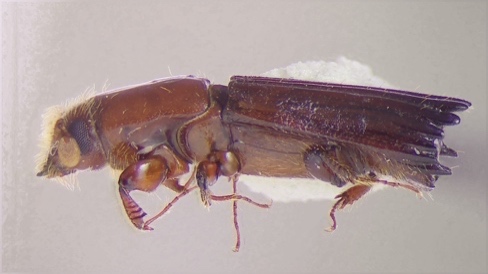** |
| 28 | Class: Insecta  Order: Coleoptera  Family: Bostrichidae  Subfamily: Bostrichinae  Género: *Xylomeira* | **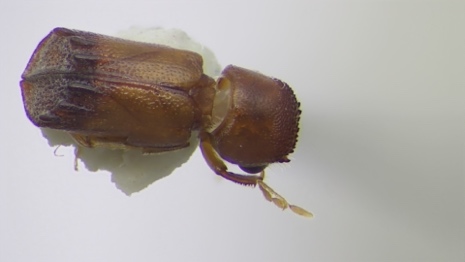** | **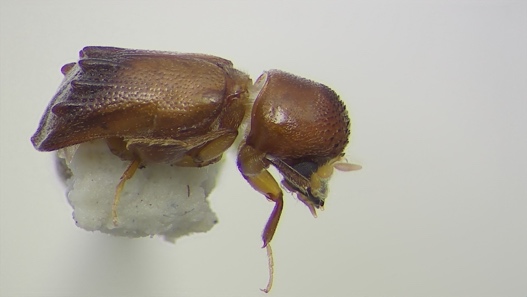** |
